# External cavity-quantum cascade laser infrared spectroscopy for secondary structure analysis of proteins at low concentrations

**DOI:** 10.1038/srep33556

**Published:** 2016-09-16

**Authors:** Andreas Schwaighofer, Mirta R. Alcaráz, Can Araman, Héctor Goicoechea, Bernhard Lendl

**Affiliations:** 1 Institute of Chemical Technologies and Analytics, Vienna University of Technology, Getreidemarkt 9/164-UPA, 1060 Vienna, Austria; 2 Laboratorio de Desarrollo Analítico y Quimiometría, FBCB, Universidad Nacional del Litoral-CONICET, Ciudad Universitaria, 3000 Santa Fe, Argentina; 3 Department of Chemistry, Institute of Biological Chemistry, University of Vienna, Währinger Straße 38, 1090 Vienna, Austria

## Abstract

Fourier transform infrared (FTIR) and circular dichroism (CD) spectroscopy are analytical techniques employed for the analysis of protein secondary structure. The use of CD spectroscopy is limited to low protein concentrations (<2 mg ml^−1^), while FTIR spectroscopy is commonly used in a higher concentration range (>5 mg ml^−1^). Here we introduce a quantum cascade laser (QCL)-based IR transmission setup for analysis of protein and polypeptide secondary structure at concentrations as low as 0.25 mg ml^−1^ in deuterated buffer solution. We present dynamic QCL-IR spectra of the temperature-induced α-helix to β-sheet transition of poly-L-lysine. The concentration dependence of the α-β transition temperature between 0.25 and 10 mg ml^−1^ was investigated by QCL-IR, FTIR and CD spectroscopy. By using QCL-IR spectroscopy it is possible to perform IR spectroscopic analysis in the same concentration range as CD spectroscopy, thus enabling a combined analysis of biomolecules secondary structure by CD and IR spectroscopy.

Structural analysis of biomolecules is of great importance in biology and biochemistry for characterising folding properties of proteins and polypeptides, as well as monitoring dynamic changes upon perturbation. Analytical methods used for investigation of biomolecule secondary structure include X-ray crystallography, nuclear magnetic resonance (NMR), circular dichroism (CD), as well as Raman and Fourier transform infrared (FTIR) spectroscopy^1^. Both NMR spectroscopy and X-ray crystallography are capable of providing structural information at atomic levels of resolution. However, NMR spectroscopy is restricted to relatively small biomolecules (≤40 kDa) at high concentrations and X-ray crystallography requires the availability of high-quality crystals of proteins, which is particularly demanding for membrane proteins^2^,^3^,^4^.

In contrast, CD, Raman and FTIR spectroscopy are considered as low resolution techniques that provide overall structural information. Due to straightforward sample preparation and fast acquisition time, these methods are routinely used for rapid determination of secondary structure of proteins and for monitoring dynamic changes of protein structure. Due to their respective characteristics, infrared (IR) spectroscopy provides more dependable estimates of antiparallel β-sheets, whereas CD spectroscopy gives more confinable predictions of α-helix structures^5^. Regarding the complementary information provided, joint application of both methods would deliver most reliable results^6^,^7^.

CD spectroscopy is a technique based on the difference in the absorption of the left- and right-handed circularly polarized light when it is in contact with the optically active compounds, or chromophores, present in the sample. In proteins, the most relevant chromophore is the amide group which absorbs in the far-UV region (180–240 nm). Their electronic transitions (n→π*, π→π*) give signals at 220 and 190 nm. The periodic alignments of the amide groups in the polypeptide backbone lead to exciton coupling interactions of these electronic transitions, which occur when a number of chromophores are located in close proximity. Consequently, shifting and splitting into multiple transitions lead to characteristic CD band shapes that can be attributed to individual protein secondary structures^8^,^9^. CD spectroscopy is only applicable for liquid phase samples and is generally used for optically clear solutions at concentration ranges below 2 mg ml^−1^ 10,11.

IR spectroscopy is a well-established analytical technique used to study the structure of polypeptides and proteins^12^, based on the absorption of IR light which induces vibrational excitations in molecules. The frequency of IR absorption is associated with the strength of the vibrating bond and the masses of the involved atoms, whereas absorption intensity is correlated with the change of the dipole moment^13^. In proteins, the vibrations of the polypeptide repeat units result in nine characteristic group frequencies referred to as amide bands. The amide I (1,600–1,700 cm^−1^) and amide II (1,500–1,600 cm^−1^) bands are the two most prominent bands in the protein IR spectrum^12^. For investigation of protein secondary structure, the amide I band is usually used, which originates from the C=O stretching and N-H in-phase bending vibration of the amide group^14^. Differing pattern of hydrogen bonding, dipole-dipole interactions, and geometric orientations in the α-helices, β-sheets, turns and random coil structures, induce different frequencies of the C=O vibrations that can be correlated with the individual secondary structural folding^15^.

IR transmission measurements are frequently employed for investigation of protein structure in solution. When working with proteins in aqueous solution, the strong absorbance of the HOH bending band of water near 1,645 cm^−1^, overlapping with the protein amide I band, requires short path lengths, typically around 8 μm for conventional FTIR spectrometers^3^,^12^. To overcome this drawback, D_2_O-based buffers are alternatively used as solvents. The DOD bending band is located at ~1,200 cm^−1^, thus not interfering with the protein amide I band. For FTIR transmission measurements of proteins in deuterated medium, peptide and protein concentrations between 7 and 15 mg ml^−1^ are commonly used^16^,^17^,^18^,^19^,^20^.

Quantum cascade lasers (QCLs) have been introduced as a mid-IR light source two decades ago. QCLs are unipolar lasers based on inter sub-band transitions of electrons within the semiconductor conduction band. They provide spectral power densities several orders of magnitude higher than thermal light sources, such as globars, which are conventionally used in FTIR spectrometers. At the beginning, QCLs were predominantly used for gas phase analysis due to their limited spectral tuning range. Since the commercial availability of external cavity-QCLs (EC-QCLs), which combine a large spectral tuning range with high spectral power densities, this type of light source has increasingly been used for studies of liquid samples^21^,^22^. Application of these high-emission power light sources has allowed to significantly increase the optical path for transmission measurements^23^. Most recently, EC-QCL-based IR transmission measurements have been accomplished for the analysis of protein secondary structure in aqueous solution^24^,^25^.

In this work, we introduce an EC-QCL-based IR transmission setup for secondary structure analysis of proteins and polypeptides in deuterated solutions at concentrations as low as 0.25 mg ml^−1^. IR spectra acquired with the laser-based setup show excellent comparability with spectra obtained by conventional FTIR spectroscopy. By example of the concentration-dependent temperature-induced α-β transition of poly-L-lysine (PLL), we demonstrate the low accessible concentration range for the EC-QCL setup, thus enabling a combined analysis of biomolecules secondary structure by CD and IR spectroscopy.

## Results

### Experimental setup for laser-based IR spectroscopy

**Figure 1:**
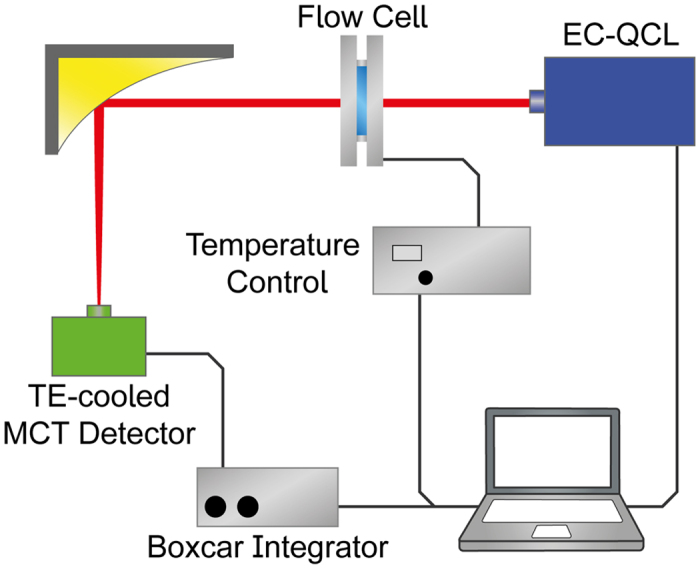
Laser-based IR spectroscopy of liquid samples. Laser-based IR transmission setup employing an EC-QCL as light source. For measurements of proteins and polypeptides in deuterated solution, a temperature-controlled flow cell with 478 μm-path length was used. The IR light is directed to the thermoelectrically cooled MCT-detector by a gold plated off-axis parabolic mirror. All elements of the setup are centrally controlled by a LabView-based GUI.

The IR spectra of proteins and polypeptides in deuterated solution were recorded by using a custom-made laser-based IR transmission setup (Fig. 1). The experimental setup is composed of an EC-QCL with a spectral tuning range covering the amide I region of proteins, a peltier-cooled MCT-detector and a temperature-controlled flow cell with a path length of 478 μm. A crucial step for enabling protein measurements employing the EC-QCL light source has been to eliminate high noise levels in the absorbance spectra. These artefacts result from mechanical imperfections in the tuning mechanism and shifts in the mode-hop fine structure of the EC-QCL emission curve within consecutive scans. For the purpose of noise reduction, we developed a data processing routine that employs the correlation optimized warping (COW) algorithm^24^. This algorithm allowed correcting mode-hop shifts across multiple scans of one single beam spectrum as well as aligning background and sample single beam spectra prior to calculating the absorbance spectrum. A further important measure to permit obtaining accurate IR spectra of proteins has been the implementation of wavenumber calibration of the laser-based setup. This procedure has proved to be necessary due to the dispersive nature of spectra acquisition. When using a light source such as an EC-QCL, one sweep of the grating over time results in the wavenumber scan. The temporal axis directly corresponds to the wavenumber axis; consequently, wavenumber inaccuracies of the EC-QCL as well as time lags introduced during data acquisition are directly translated into wavenumber deviations of the final spectra. In order to correct the deviations in the wavenumber scale, we utilized the narrow absorption lines of the ubiquitous water vapour as reference^24^.

### Protein spectra recorded with the EC-QCL-based setup

**Figure 2:**
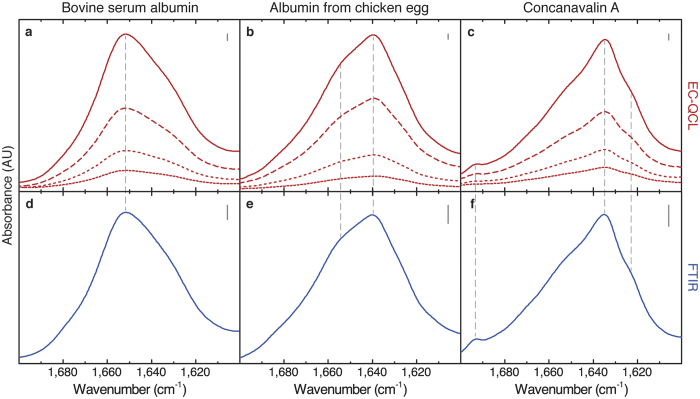
Comparison of protein IR spectra acquired by QCL-IR and FTIR spectroscopy. (**a**–**c**) IR absorbance spectra recorded with the EC-QCL-based setup of 2 mg ml^−1^ (red solid line), 1 mg ml^−1^ (red dashed line), 0.50 mg ml^−1^ (red short dashed line) and 0.25 mg ml^−1^ (red dotted line) protein solutions, and (**d**–**f**) 5 mg ml^−1^ (blue solid line) protein solution obtained by FTIR spectroscopy. Grey dashed lines highlight the excellent overlap of the protein bands in the IR spectra acquired by QCL-IR and FTIR spectroscopy. Grey bars indicate the absorbance of 10 mAU.

For the acquisition of protein spectra in deuterated solution, optimal results were obtained by using a transmission cell with 478 μm-path length^23^. Model proteins were chosen to exhibit diverse secondary structures revealing different spectral features in the IR spectrum. In Fig. 2a–c, we show IR spectra of bovine serum albumin (BSA), albumin from chicken egg (OVA) and concanavalin A (ConA) in deuterated medium acquired with the EC-QCL-based setup, at protein concentrations ranging from 2 mg ml^−1^ to as low as 0.25 mg ml^−1^. The spectrum of BSA exhibits an amide I band maximum at 1,652 cm^−1^, significant for proteins predominantly composed of α-helix secondary structure in deuterated solution^12^,^26^. OVA contains both α-helices and β-sheets in equal shares^27^, resulting in a band maximum at 1,639 cm^−1^ with a shoulder at 1,654 cm^−1 ^28. ConA mainly consists of β-sheets and displays a distinctive IR spectrum with an amide I band maximum at 1,635 cm^−1^ and sidebands at 1,693 and 1,623 cm^−1 ^29. Characteristic spectral features of the individual secondary structures could be identified at protein concentrations as low as 0.25 mg ml^−1^. For reference, FTIR spectra of the protein solutions were recorded (Fig. 2d–f). Comparison analysis between spectra reveals excellent agreement of the protein spectra recorded by using the EC-QCL-based setup with the spectra acquired by the well-established FTIR spectroscopy. In order to quantify the congruence between the spectra, the degree of spectral overlap (s_12_) was computed. Using Eq. 1, the *s*_12_ values obtained for BSA, OVA, and ConA were 0.9993, 0.9986 and 0.9934, respectively. These figures allow us to conclude that the spectra acquired with the EC-QCL setup contain equivalent information as the FTIR spectra.

### QCL-IR spectra of the heat-induced α-β transition of PLL

Poly-L-lysine is a prominent model system for the investigation of secondary structure. Its conformation can be readily altered by different types of external perturbation. When it is present as thin film, the α-β transition can be induced by decreasing the hydration of the polypeptide film^30^,^31^. In solution, its secondary structure depends on various conditions, e.g., pH value^32^, temperature^33^, pressure^34^ and chain length^35^. At atmospheric pressure and acidic to mildly basic pH, charge repulsion between the protonated lysine groups inhibits the formation of ordered structures such as regular helices or sheets, thus the polypeptide favours a random or unordered structure^34^. On the contrary, at pH values higher than its pKa (10.5), the lysine side chain is deprotonated and PLL adopts the α-helical conformation. Under these conditions, i.e., pH values higher than its pKa, upon heating to 50 °C, the structure of PLL transforms into β-sheets^32^,^33^,^35^,^36^. However, neither the low-temperature α-helical nor the high-temperature β-sheet structure are conformationally homogenous^32^. It has been demonstrated that the rate of heat-induced β-sheet formation increases with rising pH value and with increasing PLL concentration^37^. Here we employ QCL-IR, FTIR and CD spectroscopy to study the concentration dependence of the thermally-induced α-β transition of PLL in the concentration range between 0.25 and 10 mg ml^−1^.

**Figure 3:**
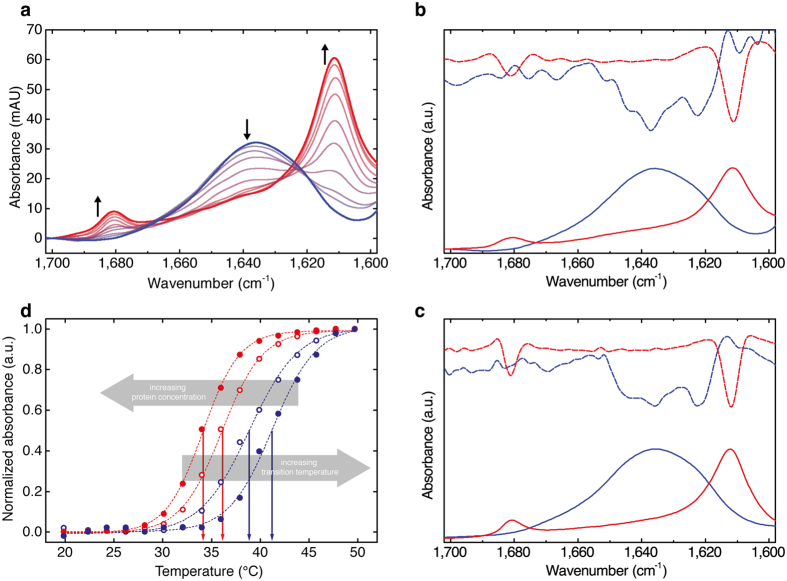
Temperature-induced conformational change of PLL monitored by IR spectroscopy. (**a**) IR spectra of 0.50 mg ml^−1^ PLL in deuterated phosphate buffer pD 12.4 recorded as function of the temperature by using the EC-QCL-based IR transmission setup. The arrows indicate directions of spectral changes with temperature. (**b**) IR spectra (solid lines) and second-derivative (dashed lines) of 0.50 mg ml^−1^ PLL in 50 mmol l^−1^ deuterated phosphate buffer pD 12.4 at 20 °C (blue) and 50 °C (red) acquired with the EC-QCL-based IR transmission setup. (**c**) IR spectra (solid lines) and second-derivative (dashed lines) of 5 mg ml^−1^ PLL in deuterated phosphate buffer pD 12.4 at 20 °C (blue) and 50 °C (red) acquired by FTIR spectroscopy. **(d)** Progression of the relative change in IR absorbance obtained by evaluation of the band height at 1,611 cm^−1^, characteristic for intermolecular, antiparallel β-sheet conformation at different temperatures (data points). Spectra at PLL concentrations of 0.50 mg ml^−1^ (filled blue circles) and 3 mg ml^−1^ (empty blue circles) were obtained with the EC-QCL-based setup; data for 6.50 mg ml^−1^ (empty red circles) and 9.50 mg ml^−1^ (filled red circles) were acquired by FTIR spectroscopy. Dashed lines represent the fitted sigmoidal curve and the arrows indicate the inflection point of the fitted curve.

Figure 3a shows the dynamic IR spectra of the temperature-induced α-β transition of PLL acquired with the EC-QCL-based setup. The spectrum recorded at 20 °C shows a band maximum at 1,635 cm^−1^, characteristic for the α-helical structure adopted by PLL at these conditions in D_2_O-based solution^32^. Compared to absorption bands typically obtained for α-helices in globular proteins (approx. 1,652 cm^−1^ in deuterated solution)^12^, the spectral position of the α-helical conformation of PLL is shifted to lower wavenumbers. This change of the band maximum has been explained by undisrupted intra- and inter-chain coupling owing to the high degree of regularity in the helix structure of a homopolypeptide^38^. Further, the precise band position depends on the chain length of the polypeptide^35^,^38^. The broad shape of the absorption band, also illustrated by the outline of the second-derivative spectra (Fig. 3b), has been associated with helices establishing unequally strong hydrogen bonds with water due to different solvent exposure^35^,^39^. Upon increasing the temperature, the band shape attributed to the α-helix configuration decreases as two bands at 1,611 and 1,681 cm^1^ emerge. This arising spectral pattern comprising of a high- and low-frequency band has commonly been attributed to intermolecular, antiparallel β-sheets in PLL^32^,^34^,^39^.

### Comparison of FTIR and QCL-IR spectra

**Figure 4:**
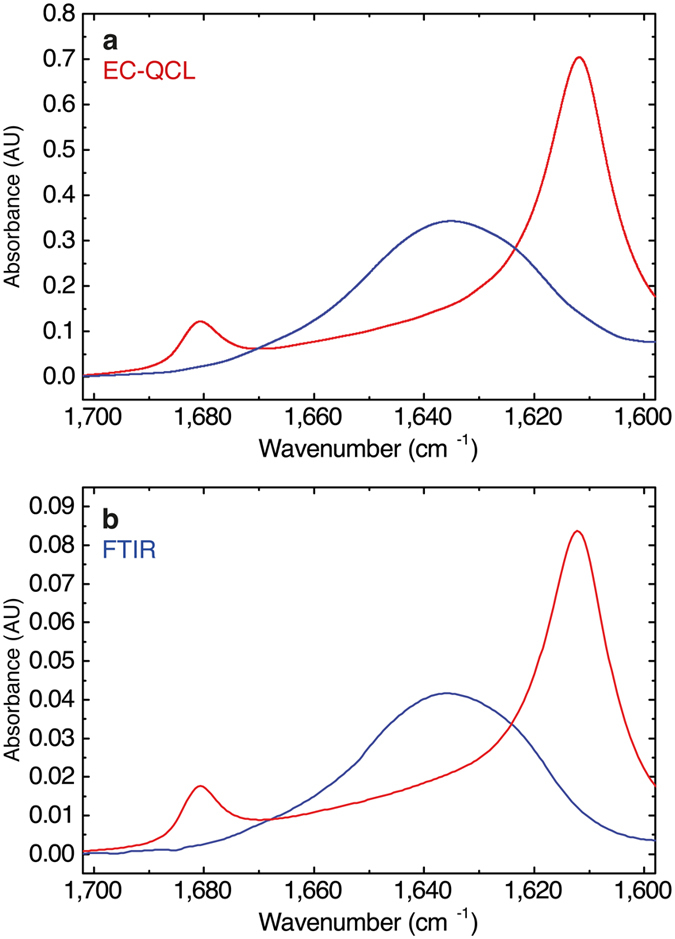
Comparison of poly-L-lysine IR spectra acquired by QCL-IR and FTIR spectroscopy. IR absorbance spectra of 5 mg ml^−1^ PLL in deuterated phosphate buffer pD 12.4 at 20 °C (blue) and 50 °C (red) recorded with (**a**) the EC-QCL-based IR transmission setup and (**b**) FTIR spectroscopy.

We performed FTIR measurements of the heat-induced PLL α-β transition at higher polypeptide concentrations to compare results with the spectra recorded with the EC-QCL-based setup by means of IR absorbance and second-derivative spectra (Fig. 3b,c). Figure 4 shows a comparison of the IR spectra of 5 mg ml^−1^ PLL acquired with the EC-QCL-based IR transmission setup and FTIR spectroscopy. Evaluation of band positions and shape of the low temperature α-helical as well as high temperature β-sheet conformation of PLL reveals high congruence between the two spectra sets. Furthermore, the degree of spectral overlap was computed in order to quantify the conformance between the IR spectra acquired with the EC-QCL setup and FTIR spectroscopy. The *s*_12_ values obtained for PLL at 20 °C and 50 °C were 0.9886 and 0.9894, respectively. The absorbance values of IR bands in the spectra obtained by the EC-QCL-based IR transmission setup are considerably higher than for the FTIR spectroscopy, owing to the larger optical path length possible to employ due to the higher emission power of the laser light source. For evaluation of the noise level, 100% lines have been evaluated, that are obtained by calculating the absorbance spectrum of two subsequent single beam spectra of the same sample at identical conditions. Under ideal conditions, the result would be a flat line at 100% transmittance, corresponding to zero absorbance^40^. The RMS (root-mean-square) of the 100% line at path lengths employed for protein measurements is 0.015 mAU and 0.121 mAU for the FTIR setup and EC-QCL setup, respectively. However, when comparing the noise levels of the two spectrometers, the difference in detectivity between the employed detectors needs to be considered, which is 10 times higher for the LN_2_ cooled MCT-detector in the FTIR spectrometer compared to the Peltier cooled MCT-detector used in the QCL setup. Supplementary Fig. S1 shows a comparison of IR spectra at protein concentrations of 0.25 mg ml^−1^ acquired by the EC-QCL setup and FTIR spectroscopy at similar measurement times.

In order to compare the progress of the conformational transition at different polypeptide concentrations, we evaluated the absorbance of the low-frequency β-sheet band at 1,611 cm^−1^ at every temperature (Fig. 4a). Because the heating rate affects the α-β transition as a function of temperature^32^, the same rate was employed for QCL-IR and FTIR measurements. Values obtained from the aforementioned evaluation were fitted with a Boltzmann function for sigmoidal curves, whose inflection point represents the α-β transition temperature of the polypeptide.

For evaluation of the reproducibility of the QCL-IR and FTIR technique for monitoring the α-β transition, a triplicate of 2 mg ml^−1^ (QCL-IR) and 5 mg ml^−1^ (FTIR) PLL solution was measured. The coefficients of variation of the transition temperature at these conditions were determined to be 0.48% and 0.57% for QCL-IR and FTIR, respectively, demonstrating the excellent reproducibility of the methodology. Qualitative and quantitative comparison confirm our strong confidence in the equivalency of the IR spectra acquired with the laser-based setup with conventional FTIR instrumentation, even at low protein or polypeptide concentrations in deuterated solution.

When comparing the results obtained for the experiments performed at different PLL concentrations, we observed higher transition temperatures at low PLL concentrations, indicating lower propensity for thermally-induced β-sheet aggregation at lower polypeptide concentration (Fig. 3d).

### CD spectra of the heat-induced conformational change in PLL

**Figure 5:**
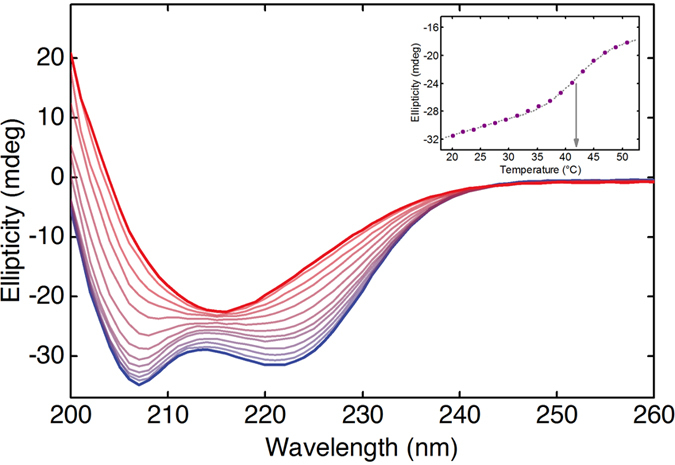
CD spectra of PLL recorded as function of temperature. Far-UV CD spectra of 0.25 mg ml^−1^ PLL in deuterated phosphate buffer pD 12.4, recorded in a temperature range between 20 and 50 °C, at 2 °C steps. At low temperatures (blue), the typical CD pattern associated with α-helical structures is present. Upon increase of the temperature, the spectral shape changes until it reaches the CD profile characteristic for β-sheet structures. Inset: Progression of the ellipticity at 222 nm with increasing temperatures (data points). Grey dotted line shows the fitted sigmoidal line and the arrow indicates the transition temperature.

For comparing the results of the newly-introduced laser-based IR transmission setup at low polypeptide concentration with an established experimental technique, we monitored the temperature-induced conformational transition of PLL by far-UV CD spectroscopy (exemplary CD spectra are shown in Fig. 5). To ensure comparability with the results obtained by IR spectroscopy, the same heating rate was applied for the CD measurements. At low temperatures, the CD signal features two negative peaks centered at 208 and 222 nm, clearly indicating the α-helical structure of PLL under these experimental conditions. Upon temperature increase, a loss of negative ellipticity could be observed at the aforementioned wavelengths. At high temperatures, the CD spectrum shows a single negative peak at 218 nm, characteristic for β-sheet structures^9^. The temperature-induced conformational change of the polypeptide was followed by evaluation of the CD signal at 222 nm, which is particularly sensitive to changes of the secondary structure^2^. As for IR measurements, the data points were fitted following a sigmoidal Boltzmann function in order to estimate the inflection point of the α-β transition temperature (Fig. 5 inset). The transition temperatures evaluated by CD spectroscopy at low PLL concentrations show excellent agreement with findings from QCL-IR spectroscopy.

### Concentration dependence of PLL α-β transition temperature

**Figure 6:**
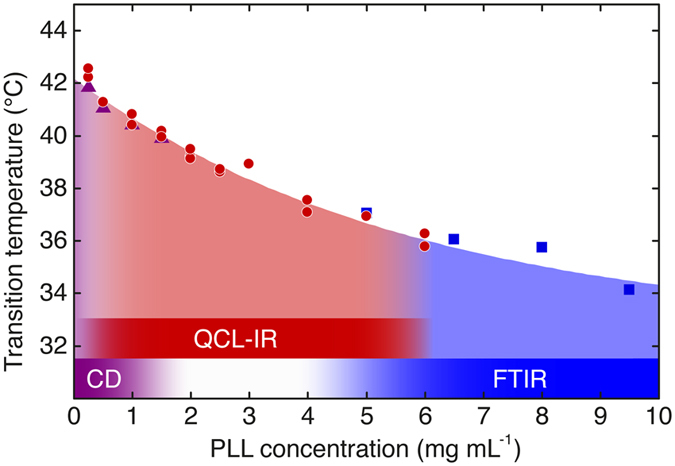
Influence of polypeptide concentration on the α-β transition temperature. Transition points of temperature-induced conformational change of α-helix to β-sheet secondary structure as determined by far-UV CD (purple triangles), QCL-IR (red circles) and FTIR (blue squares) spectroscopy. The coloured bars represent the concentration regions covered by the individual analytical techniques.

The temperature-induced conformational change of PLL was studied in the concentration range between 0.25 and 10 mg ml^−1^. Figure 6 shows the transition temperatures of the α-β transition determined by QCL-IR, FTIR and CD spectroscopy as described above. This presented, greatly congruent trend of the measured data points attests the consistency of the results obtained by three independent experimental techniques and reveals a dependence of the transition temperature on PLL concentration. The α-β transition temperature shows an exponential decay as a function of the polypeptide concentration. A comparable concentration dependence of thermally-induced conformational changes has been reported for several systems^41^,^42^,^43^. For PLL, differences in the temperature-induced α-β transition depending on polypeptide concentration have been mentioned in earlier reports^37^,^44^,^45^,^46^, but, to the best of our knowledge, it has not been investigated in a systematic manner. Here, this condition poses an excellent example to corroborate the demand for lower concentration limits and a larger accessible concentration range for protein studies using IR spectroscopy.

The thermally-induced transition of PLL values from α-helix to β-sheet at high pH involves an intermediate conformation that has been attributed to extended helices^33^,^35^ or random coil conformation^37^. In a first step, upon increasing the temperature, intramolecular hydrogen bonds stabilizing the helix configuration break and PLL reversibly adopts this random structure. Further, increasing thermal motion facilitates hydrophobic interactions between lysine side groups, stimulating β-sheet formation, which is irreversible^32^,^47^. The higher intensity of hydrophobic interactions at elevated temperatures is the basis for the increased propensity for β-sheet formation under these conditions^47^,^48^. The positive enthalpy necessary for breaking the hydrogen bonds and association of the lysyl residues forming the β-structure is provided by the heating process^37^. Finally, the concentration dependence of the α-β transition as observed in this study can be explained by the intermolecular nature of β-sheet formation in PLL^37^,^49^.

## Discussion

Here, we introduced an EC-QCL-based IR transmission setup for the investigation of biomolecule secondary structure in deuterated solution. To showcase the potential of this novel setup, we followed dynamic conformational changes at polypeptide concentrations, which had not been previously reported by FTIR spectroscopy, to the best of our knowledge.

QCL-IR measurements at biomolecule concentrations as low as 0.25 mg ml^−1^ allow the combination of CD and IR spectroscopy, which is beneficial for comprehensive understanding of complex biological samples^6^. Particularly for systems that show concentration-dependent effects, a large available concentration range is of major importance. It has been demonstrated that this can be achieved by the employed EC-QCL based IR transmission setup. Further highlights of the setup include its miniaturized layout. Both the laser light source as well as the detector are thermoelectrically-cooled, enabling room-temperature operation without need for cooling with liquid nitrogen. Moreover, the high achievable path-length compared to conventional FTIR spectroscopy adds to the ruggedness of the analysis system. In this respect, we envision that laser-based IR light sources fundamentally change the problem solving capabilities of mid-IR biospectroscopy. Even though they are still in the infancy of their evolution, broadly tunable EC-QCLs have already found broad application for analysis of biological samples^30^,^50^,^51^,^52^,^53^,^54^. Regarding transmission spectroscopy of biomolecules, we foresee manyfold applications in studies that involve samples whose protein concentrations are intrinsicly limited, e.g., in body fluids or in conformational investigations where particularly low protein concentrations are relevant, such as in vaccine studies or poorly soluble protein therapeutics^55^.

## Methods

### Materials

Sodium phosphate tribasic anhydrous, tech. (Na_3_PO_4_) was purchased from Alfa-Aesar (Karlsruhe, Germany). Deuterium oxide (99.9 atom % D, D_2_O), deuterium chloride solution 35 wt. % in D_2_O (99 atom % D, DCl), sodium deuteroxide 30 wt. % in D_2_O (99 atom % D, NaOD), albumin from chicken egg white (OVA), concanavalin A from *Canavalia ensiformis* (Jack bean) type IV (ConA), bovine serum albumin (≥98.0%, BSA) and α-poly-L-lysine hydrobromide (mol wt. 15,000–30,000 by viscosity, PLL) were obtained by Sigma-Aldrich (Steinheim, Germany) and used as purchased.

### Sample preparation

50.0 mmol l^−1^ and 10.0 mmol l^−1^ deuterated phosphate buffer solutions were prepared by dissolving the appropriate amount of Na_3_PO_4_ in D_2_O, and adjusting the pH (corresponding to pD = pH + 0.4)^56^ with DCl or NaOD.

Protein stock solutions were daily prepared by dissolving 10.0 mg of protein powder directly in 1.00 ml of 50 mmol l^−1^ deuterated phosphate buffer pD 7.0 for BSA and OVA, and pD 6.2 for ConA. A set of four samples for each protein was prepared by transferring appropriate aliquots of the stock solution and completing the volume to 1.00 ml with the corresponding buffer solution. For QCL-IR measurements, the final protein concentrations were ranging between 0.25 and 2 mg ml^−1^. Further, a 5 mg ml^−1^ protein solution was prepared for FTIR measurements.

The stock solution of PLL was daily prepared by dissolving 10.0 mg of PLL powder directly in 1.00 ml of deuterated phosphate buffer pD 12.4. PLL sample solutions were prepared by transferring appropriate aliquots of PLL stock solution and completing the volume to 1.00 ml with deuterated phosphate buffer pD 12.4. The pD of every PLL sample solution was verified after preparation and adjusted, if necessary, with NaOD to 12.4. For CD measurements, sample solutions were prepared at polypeptide concentrations of 0.25, 0.50, 1.00 and 1.50 mg ml^−1^ in 10.0 mmol l^−1^ deuterated phosphate buffer. For these measurements, lower buffer concentration was used as for IR measurements, because phosphate ions show considerable absorbance below 210 nm^2^. Preliminary experiments have proved that the difference in buffer concentration does not have effect on the transition temperature of the polypeptide. For QCL-IR measurements, the PLL samples were prepared with final concentrations ranging between 0.25 and 6 mg ml^−1^ in 50.0 mmol l^−1^ phosphate buffer. Finally, samples with PLL concentrations of 5.00, 6.50, 8.00 and 9.50 mg ml^−1^ were prepared in 50.0 mmol l^−1^ deuterated phosphate buffer for FTIR analysis. In all cases, the respective phosphate buffer solutions were taken as background and corresponding spectra were recorded under identical conditions as sample spectra.

Sample preparation was performed under a dry nitrogen stream inside a glove box. pD measurements were carried out with a pH 330i (Wissenschaftlich-Technische Werkstätten GmbH, Weilheim, Germany) potentiometer equipped with a Sentix^®^ MicD (Wissenschaftlich-Technische Werkstätten GmbH, Weilheim, Germany) combined glass electrode and temperature probe.

### QCL-IR measurements

The measurements were performed on a custom-made EC-QCL-based IR transmission setup equipped with an external cavity-quantum cascade laser (spectral tuning range = 1,729.30–1,565.06 cm^−1^; Daylight Solutions Inc., San Diego, USA), a temperature-controlled flow cell, and a thermoelectrically cooled (−60 °C) MCT-detector (MCT-7-TE3; D* = 4 × 10^9^ cm Hz^0.5^ W^−1^ at 9.2 μm; Infrared Associates Inc., USA). The laser was thermoelectrically cooled (head temperature = 18 °C) and operated in pulsed mode at a repetition rate of 100 kHz and a pulse width of 500 ns. A two-channel boxcar integrator was used to process the measured signal before digitization by a NI DAQ 9239 24-bit ADC (National Instruments Corp., Austin, USA). The whole setup was controlled by a LabView-based GUI 11.0 (National Instruments Corp., Austin, USA, 2011) with server-client program structure. A custom-made temperature-controlled flow cell equipped with two MIR transparent 2 mm CaF_2_ windows and a 478 μm-thick PTFE spacer was used to perform the measurements. To reduce the influence of water vapour, the setup was placed in a housing of polyethylene foil and constantly flushed with dry air. The spectral resolution of the EC-QCL setup was determined to be 0.2 and 1.2 cm^−1^ for nonfiltered and filtered spectra, respectively^24^.

The temperature-controlled experiments were carried out with a custom-made temperature cell consisting of nine thermoelectric cooling (TEC) elements, stabilized by liquid water. For PLL measurements, a total of 20 scans were recorded for background and sample single beam spectra (total measurement time = 100 s) in a temperature range of 20–50 °C (ΔT = 2 °C). Prior to data acquisition, the cell was equilibrated for 240 s at each temperature step. For protein measurements, a total of 100 scans were acquired for background and sample single beam spectra at 25.0 °C. In order to minimize spectral noise resulting from mechanical imperfections of the tuning mechanism of EC-QCL light source, a data processing routine based on correlation optimized warping (COW) algorithm was applied. For wavenumber accuracy, the EC-QCL-based setup was calibrated using the absorption bands of water vapour^24^.

In order to quantitatively evaluate the comparability of the IR absorbance spectra acquired by EC-QCL and FTIR spectroscopy by the degree of spectral overlap (*s*_12_) between FTIR (**s**_1_) and EC-QCL (**s**_2_), the following expression was employed^57^:


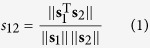


The value of *s*_12_ ranges from 0 to 1, corresponding to no overlapping and complete overlapping, respectively.

Data processing and analysis were performed in MATLAB R2014b (MathWorks, Inc., Natick, MA, 2014).

### FTIR measurements

The measurements were performed using a Vertex 80v FTIR spectrometer (Bruker Corp., Ettlingen, Germany) equipped with a liquid nitrogen cooled MCT-detector (D* = 4 × 10^10^ cm Hz^0.5^ W^−1^ at 9.2 μm). The custom-made temperature-controlled cell was equipped with a 53 μm-thick PTFE spacer. The sample compartment of the spectrometer was continuously flushed with dry air during IR measurements. IR spectra were acquired with a spectral resolution of 2 cm^−1^ in double-sided acquisition mode using a Blackman-Harris 3-term apodization function and a zero filling factor of 2. For PLL measurements, a total of 450 scans were averaged per spectrum (total measurement time = 100 s), acquired in a temperature range of 20–50 °C (ΔT = 2 °C). After setting the temperature, the cell was allowed to equilibrate for 240 s prior to spectrum acquisition. Following this procedure, the heating rate was the same as for QCL-IR measurements. Protein measurements were carried out at 25.0 °C and a total of 64 scans were averaged per spectrum. Spectra analysis was performed by using the software package OPUS 7.2 (Bruker Corp., Ettlingen, Germany). If necessary, absorption bands of water vapour in the atmosphere were subtracted.

### CD measurements

Far-UV CD measurements were carried out using a Chirascan-plus CD spectrophotometer (Applied Photophysics Ltd., Leatherhead, Surrey, UK) equipped with a peltier temperature controller unit (TC125; Quantum Northwest Inc., Liberty Lake, WA, USA) in a 1 mm-quartz cell. The spectra were registered every 1 nm in the spectral range of 200–260 nm (total measurement time = 100 s), with an acquisition time of 1 s per point, in the temperature range of 20–50 °C (ΔT = 2 °C), after an equilibration time of 240 s at each temperature step, in agreement of the heating rate and total measurement time as for IR measurements.

## Additional Information

**How to cite this article**: Schwaighofer, A. *et al*. External cavity-quantum cascade laser infrared spectroscopy for secondary structure analysis of proteins at low concentrations. *Sci. Rep.* **6**, 33556; doi: 10.1038/srep33556 (2016).

## Supplementary Material

Supplementary Information
