# Supplementary material for: External cavity-quantum cascade laser infrared spectroscopy for secondary structure analysis of proteins at low concentrations
Source: Sci Rep. 2016 Sep 16;6:33556. doi: 10.1038/srep33556 (PMC5025714; doi:10.1038/srep33556)
Supplement: Supplementary Information [file srep33556-s1.pdf]

Supplementary Material for

External cavity-quantum cascade laser infrared spectroscopy for secondary structure analysis of proteins at low concentrations

Andreas Schwaighofer[1,†], Mirta R. Alcaráz[1,2,†], Can Araman[3], Héctor Goicoechea[2] & Bernhard Lendl[1,*]

[1]Institute of Chemical Technologies and Analytics, Vienna University of Technology, Getreidemarkt 9/164-UPA, 1060 Vienna, Austria.

[2]Laboratorio de Desarrollo Analítico y Quimiometría, FBCB, Universidad Nacional del Litoral-CONICET, Ciudad Universitaria, 3000 Santa Fe, Argentina.

[3]Department of Chemistry, Institute of Biological Chemistry, University of Vienna, Währinger Straße 38, 1090 Vienna, Austria

[†] These authors contributed equally to this work.

* Corresponding Author: Bernhard Lendl
bernhard.lendl@tuwien.ac.at

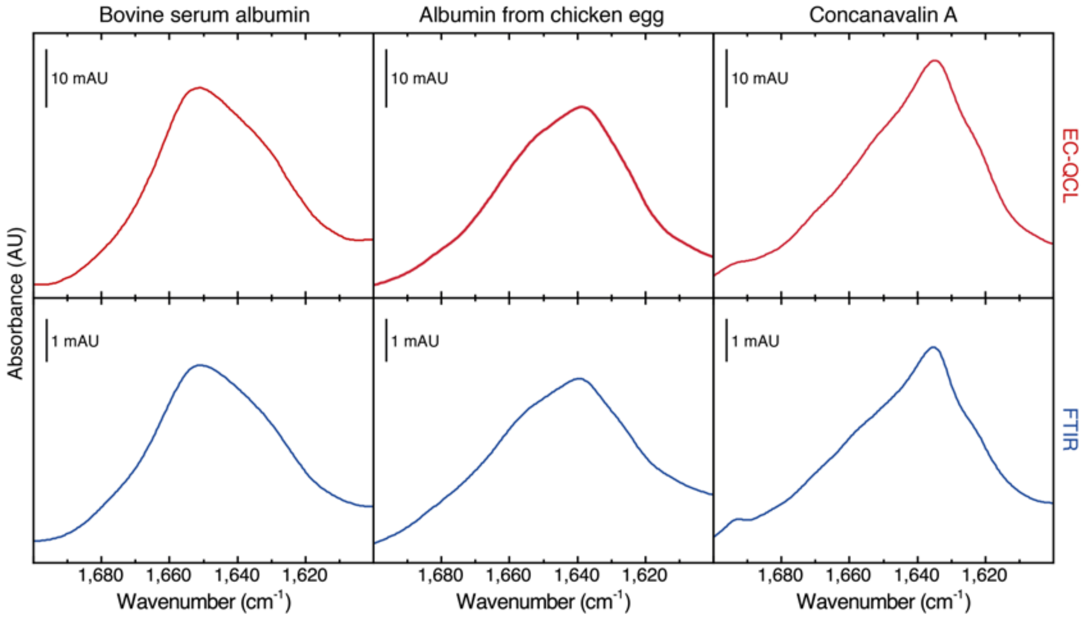

Supplementary Fig. S1. IR absorbance spectra recorded of 0.25 mg ml^{-1} protein solutions with the EC-QCL-based setup (red solid line) and FTIR spectroscopy (blue solid line) acquired at similar measurement times (500 s), equivalent to 100 and 2400 scans for the EC-QCL-based setup and the FTIR spectrometer, respectively.
